# Linderae Radix Ethanol Extract Alleviates Diet-Induced Hyperlipidemia by Regulating Bile Acid Metabolism Through gut Microbiota

**DOI:** 10.3389/fphar.2021.627920

**Published:** 2021-02-17

**Authors:** Tao Jiang, Chuyun Xu, Huifang Liu, Muyi Liu, Minmin Wang, Jiarui Jiang, Guangji Zhang, Chuqi Yang, Jianbo Huang, Zhaohuan Lou

**Affiliations:** ^1^ School of Basic Medical Sciences, Zhejiang Chinese Medical University, Hangzhou, China; ^2^ College of Pharmaceutical Sciences, Zhejiang Chinese Medical University, Hangzhou, China; ^3^ Biological Sciences Department, Computer Science Department, Purdue University, West Lafayette, IN, United States

**Keywords:** natural drug, hyperlipidemia, bile acid metabolism, gut microbiota, lipid metabolism, enterohepatic circulation

## Abstract

Hyperlipidemia is a common metabolic disorder and regarded as one of the main risk factors for cardiovascular disease. The gut microbiota has been identified as a potential contributor to hyperlipidemia as it can greatly regulate bile acid metabolism. Linderae radix is a natural medicine widely used in the treatment of a variety of diseases and is also a common drug for hyperlipidemia. Recently, the lipid-lowering effect of Linderae radix are receiving increasing attention but the underlying mechanism remains unknown. The study aimed to investigate the effects of Linderae radix ethanol extract (LREE) on gut microbiota in rats with hyperlipidemia syndrome. We established a hyperlipidemia rat model using a high-fat diet and used LREE as the intervention. Blood lipid levels and pathological examination were measured to assess the effects of LREE on hyperlipidemia. The gut microbiota was determined by 16s rDNA sequencing and the bile acid metabolism-related proteins were detected by western blot to discover the underlying correlations. The results show that LREE lowered TC, TG, and LDL levels effectively, and it also alleviated liver injury by reducing ALT and AST activity. Meanwhile, LREE improved gut microbiota disturbance caused by HFD via increasing intestinal microbiota diversity and changing the abundance of the Firmicutes, Bacteroidetes, and Actinobacteria. In addition, LREE can increase bile acid reabsorption and promote fecal excretion through farnesoid X receptor (FXR), apical sodium-dependent bile acid transporter (ASBT), organic solute transporter alpha (OST-α), and cytochrome P450 family 7 Subfamily A Member 1 (CYP7A1) thus restoring abnormal bile acid metabolism caused by hyperlipidemia.

## Introduction

Hyperlipidemia is a health problem characterized by a high level of lipids, including cholesterol and triglycerides in the serum. About 41.2% of adults aged 40–64 have elevated LDL levels (Pencina et al., 2014) and are at increased risk of developing hyperlipidemia. Hyperlipidemia is the main risk factor of cardiovascular disease (CVD) (Subcommittee, 2016) and large clinical trials have proved that lowering LDL-C levels reduce cardiovascular events and mortality rate (Mihaylova et al., 2012; Bibbins-Domingo et al., 2016). Currently, statins are the major medications to treat hyperlipidemia but side effects including muscle myopathy and derangements in hepatic function (Maggo et al., 2011) limit its clinical application. In addition, fibrates, ezetimibe, bile sequestrants, and niacin are common drugs for hyperlipidemia but the application of them is still limited because of the clinical effects and side effects. Therefore, it is necessary to identify new drugs for hyperlipidemia treatment. Traditional Chinese herbs have great potential for the treatment of hyperlipidemia and more than 50 patented forms of Chinese patent medicines have been approved by China Food and Drug Administration (Xie et al., 2012) as adjuvant treatment. Not only that, studies have shown that a variety of single herbs, including AlismatisRhizoma, CoptidisRhizoma, Crataegi Fructus have a good therapeutic effects on hyperlipidemia (Sham et al., 2014). Linderae radix, a traditional Chinese medicine arises from the dried root of Lindera aggregata (Sims) Kosterm, is frequently used for abdominal pain, frequent urination, and digestive system disease (Cheng et al., 2007; Huan et al., 2017). Pharmacological investigations have revealed Linderae radix to have various biological activities, including anti-inflammation, analgesia (Wang et al., 2007a; Luo et al., 2010), prevention of liver injury (Yamahara et al., 1983), and improvement of insulin resistance (Wang et al., 2010). Our previous study has proved that Linderae radix ethanol extract (LREE) can improve the symptoms and delay the progress of hyperlipidemia (Ye et al., 2020). However, the underlying mechanism remains largely unknown.

Crosstalk between gut microbiota and bile acids is an important cause of hyperlipidemia. Gut microbiota contributes to the metabolic health of the human host and, when aberrant, may lead to the occurrence of the pathogenesis of various common metabolic disorders (Fan and Pedersen, 2020). Microbiota can quickly respond to changing diets (David et al., 2014), affecting lipid absorption. In addition, they participate in the regulation of lipid metabolism-related genes and signaling pathways by producing different metabolites and affecting intestinal permeability (Schoeler and Caesar, 2019; He and You, 2020). Several studies also demonstrated that gut microbiota can intervene in lipid metabolism by affecting energy metabolism and fat accumulation (Bäckhed et al., 2004), inducing chronic inflammation (Xu et al., 2003), and regulating capacity balance (Janero et al., 2011). The diverse biological functions of gut microbiota enable it to play a key role in metabolic diseases. The bile acids are a well-known major regulator of serum cholesterol homeostasis. The imbalance between the amount of bile acid produced in the liver and the amount of bile acid excreted results in the difference between cholesterol absorption and excretion (Jia et al., 2020). Bile acids aid in the emulsification and absorption of dietary lipids (Schoeler and Caesar, 2019). Different types of bile acids also act as signaling molecules to regulate lipid levels of the host through binding to the numerous target proteins such as nuclear receptor farnesoid X receptor (FXR) and takeda G-protein coupled bile acid receptor (TGR5) (Parks et al., 1999). Since the existence of the enterohepatic circulation of bile acid, the microbial homeostasis is closely related to bile acid metabolism which further leads to differences in cholesterol absorption and excretion. In addition, gut microbiota can regulate bile acid metabolism and affect multiple bile acid receptor signaling pathways (Massafra et al., 2018). The complex relationship between them is a major contributor to the generation of metabolic diseases including hyperlipidemia. As gut microbiota and bile acid are important factors affecting lipid metabolism, in this study, we aim to study the effect of LREE on gut microbiota and bile acid in hyperlipidemia rats.

## Materials and Methods

### Preparation of LREE

**FIGURE 1:**
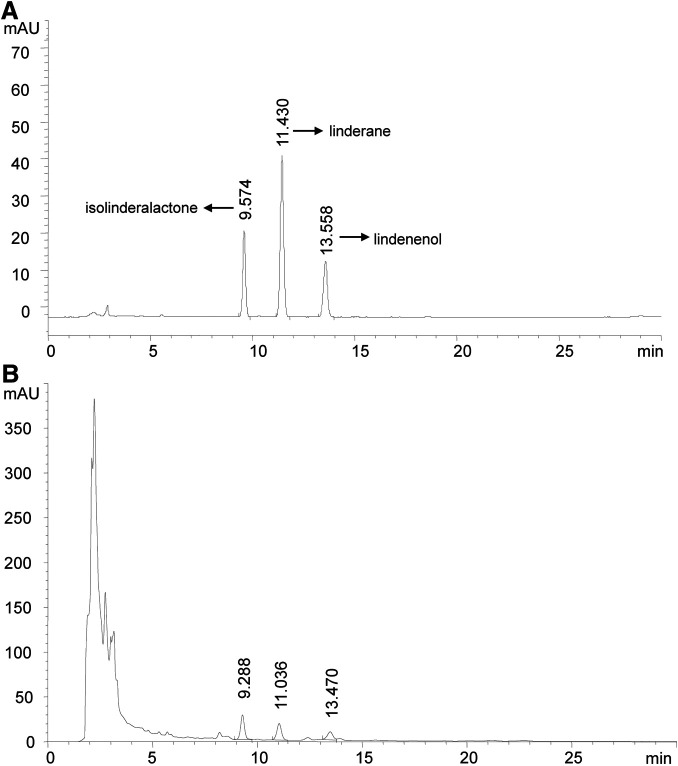
High-performance liquid chromatography chromatogram (HPLC). Isolinderalactone shows the earliest peak time. Therefore, the description of FIGURE 1A needs to be modified as follows. **(A)** The HPLC profile of standard isolinderalactone, linderane,and lindenenol are shown with a peak of 9.574, 11.430, and 13.558 min, respectively. **(B)** The HPLC profile of LREE is presented with a peak at 9.288, 11.036, and 13.470 min. They had similar retention times, which showed they might be homologs.

Linderae Radix (170301, Zhejiang Tiantaishan Wuyao Biological Engineering Co. Ltd, China) was stored at 4°C protected from light and then used to extract LREE. Briefly, 200 g Linderae Radix aggregate comminution was immersed in 1,600 ml 70% (v/v) ethanol for 90 min. Then, ethanol extract was collected and another 1,600 ml ethanol was used for a second extraction. All the filtered extract solutions were collected and then rotary evaporated into paste with 30 r/min at 45°C. The resulting pastes were dissolved in pure water to obtain 200 ml of LREE liquid finally and its quality is monitored by reversed-phase high-performance liquid chromatography (RP-HPLC) with a diode array detector (DAD) at the wavelength of 235 nm, flow rate as 1.0 ml/min, and the column temperature set as 30°C (Figure 1). LREE was stored protected from light at 4°C until further use.

### Animals and Treatment

Six-week-old Sprague-Dawley (SD) rats were provided by the Animal Supply Center of Zhejiang Academy of Medical Science [Hangzhou, China; certificate no. SCXK (Zhe) 2014–0001]. Animal procedures were carried out following the United Kingdom. Animals (Scientific Procedures) Act, 1986, and associated guidelines and examined by the Medical Ethics Committee of Zhejiang Chinese Medical University. Every three rats were housed in one cage with a comfortable environment of 25 ± 1°C, 50–60% humidity, and 12 h/12 h dark/light cycle. After one-week habituation, eight rats were assigned into the normal control (NC) group and fed a normal diet. 24 rats were assigned into three groups as HFD, Ezetimibe (EZE, 7EZPA22002, Merck Sharp and Dohme CORP), and LREE, and fed a high-fat, high-cholesterol diet (HFD, TP-2004; Components: 64% standard fodder, 20.0% sucrose, 15% Lard, 0.8% cholesterol, and 0.2% sodium cholate; Trophic Animal Feed High-tech Co. Ltd, China). At the same time, NC and HFD groups were orally administered distilled water, whereas the EZE and LREE were administered with 1 g/kg EZE and 1 g/kg LREE. Bodyweight of each rat was recorded every three days. At the end of the experiment, the fecal sample of each rat was collected respectively for intestinal microbial analysis, and then all the rats were anesthetized and sacrificed after anesthetizing. Blood, liver, and small intestine were harvested and used for the corresponding detection.

### Biochemical Assay

Blood samples were collected and left to stand for 30 min at room temperature. Then centrifugation was operated at 1,200 × g for 15 min at 4°C to separate the serum. Serum TC, TG, HDL-C, LDL-C, ALT, and AST levels were measured using a fully automatic blood biochemistry analyzer (TBA-40FR; Toshiba Medical Systems Corporation, Otawara, Japan). The liver homogenate was prepared by grinding 0.5 g of liver tissue and then centrifuged at 1,500 ×g for 10 min to separate the supernatant. Levels of TC, TG, and TBA in the liver were measured using ELISA kits and a PowerWave 340 microplate reader (BioTek Instruments, Inc., Winooski, VT, United States).

### Quantitation of Bile Acids

Comprehensive profiling and quantitation of bile acids in serum were performed by Metabo-Profile Inc (Shanghai, China) using the previously published methods with minor modifications (Xie et al., 2015, Xie et al., 2016).

### Histopathological Analysis

The liver specimens were dehydrated, embedded in paraffin, cut into 4–6 µm sections and stained using H&E for morphological observation. Lipid deposition in liver was observed by oil red O staining. Frozen liver sections were air-dried at room temperature for 2 h and fixed with 4% paraformaldehyde for 5 min. The sections were washed and then rinsed in 60% isopropanol to remove excess water. Then sections were incubated in Oil Red O reagents for 10  min, followed by the removal of Oil Red O solution with 60% isopropanol. Nuclei were stained with hematoxylin for 5 min and washed with water.

### 16S rDNA Bioinformatics Analysis and Statistics

Fecal sample pretreatment and DNA genome extraction and identification: 1 g of fecal sample was added with 30 ml of sterile PBS and then centrifuged at 1,000 rpm for 5 min to collect the precipitate. After 3 repetitions, the precipitate was resuspended with 10 ml PBS, and the precipitate was collected. Microbial community genomic DNA was extracted from the above samples using the E. Z.N.A.^®^ soil DNA Kit (Omega Bio-Tek, Norcross, GA, United States) according to manufacturer’s instructions. Genomic DNA was detected by 1.0% agarose gel electrophoresis. The V3-V4 region of 16S ribosomal RNA gene was selected for amplification with a primer of 338F (ACTCCTACGGGAGGCAGCAG)/806R (GGACTACHVGGGTWTCTAAT) (Lee et al., 2012) by an ABI GeneAmp^®^ 9700 PCR thermocycler (ABI, CA, United States). PCR products were detected by 2% agarose gel electrophoresis. Biterminal sequencing was conducted on an Illumina MiSeq PE300 platform/NovaSeq PE250 platform (Illumina, San Diego, United States) based on the standard protocols by Majorbio Bio-Pharm Technology Co. Ltd (Shanghai, China). The raw 16S rRNA gene sequencing reads were quality-filtered by Fastp v.0.20.0 (Chen et al., 2018) and merged by Flash v.1.2.7 (Magoč and Salzberg, 2011). 300 bp reads were truncated over a 50 bp sliding window, and the truncated reads shorter than 50 bp and ambiguous characters were discarded. Samples were distinguished according to the barcode and primers with exact barcode matching and nucleotide mismatch in primer matching. Operational taxonomic units (OTUs) clustering analysis of non-repetitive sequences (without single sequences) were defined with a similarity ≥ 97% (Edgar, 2013). The taxonomy of each OTU representative sequence was analyzed by RDP Classifier version 2.2 (Wang et al., 2007b) against the 16S rRNA database Silva v138 by a threshold of 0.7. The data were further analyzed using a free online tool of Majorbio Cloud Platform (www.majorbio.com).

### Western Blot analysis of bile acid metabolism related proteins

The nuclei of intestinal tissue were treated with NE-PER™ Nuclear and Cytoplasmic Extraction Reagents. In brief, the intestinal tissue was ground and rinsed, and the cytoplasmic protein and nuclear protein were extracted respectively. The BCA method was used to determine the protein concentration. Western blotting was performed according to a standard method. The dilution ratio of the primary antibody is shown in Table 1. Western blot band intensities were analyzed using ImageJ software V1.6.0 (W.S.Rasband, NIH, Bethesda, United States).

**TABLE 1:** Primary antibody information in Western Blotting experiment.

Antibody name	Brand and product number	Dilution degree	Molecular weight (kDa)
ASBT	Abcam ab82170	1:1000	38
FGF 15	Abcam ab229630	1:3000	25
FXR1	Abcam ab155124	1:5000	70
OSTα	Biorbyt orb325819	1:1000	38
CYP7A1	Biorbyt orb539102	1:1000	57
CYP8B1	Abcam ab191910	1:1000	58
GAPDH	Abcam ab181602	1:10000	36

### Statistical Analysis

Data were presented as the mean ± standard deviation. SPSS 19.0 was employed for data analyses and GraphPad Prism 8.0 was used for figure editing. Differences between two groups were analyzed by unpaired *t*-test and differences between multiple groups were analyzed by variance analysis. *p* < 0.05 was considered statistically significant.

## Results

### LREE Improves Serum Lipid Levels in Hyperlipidemia Rats

**FIGURE 2:**
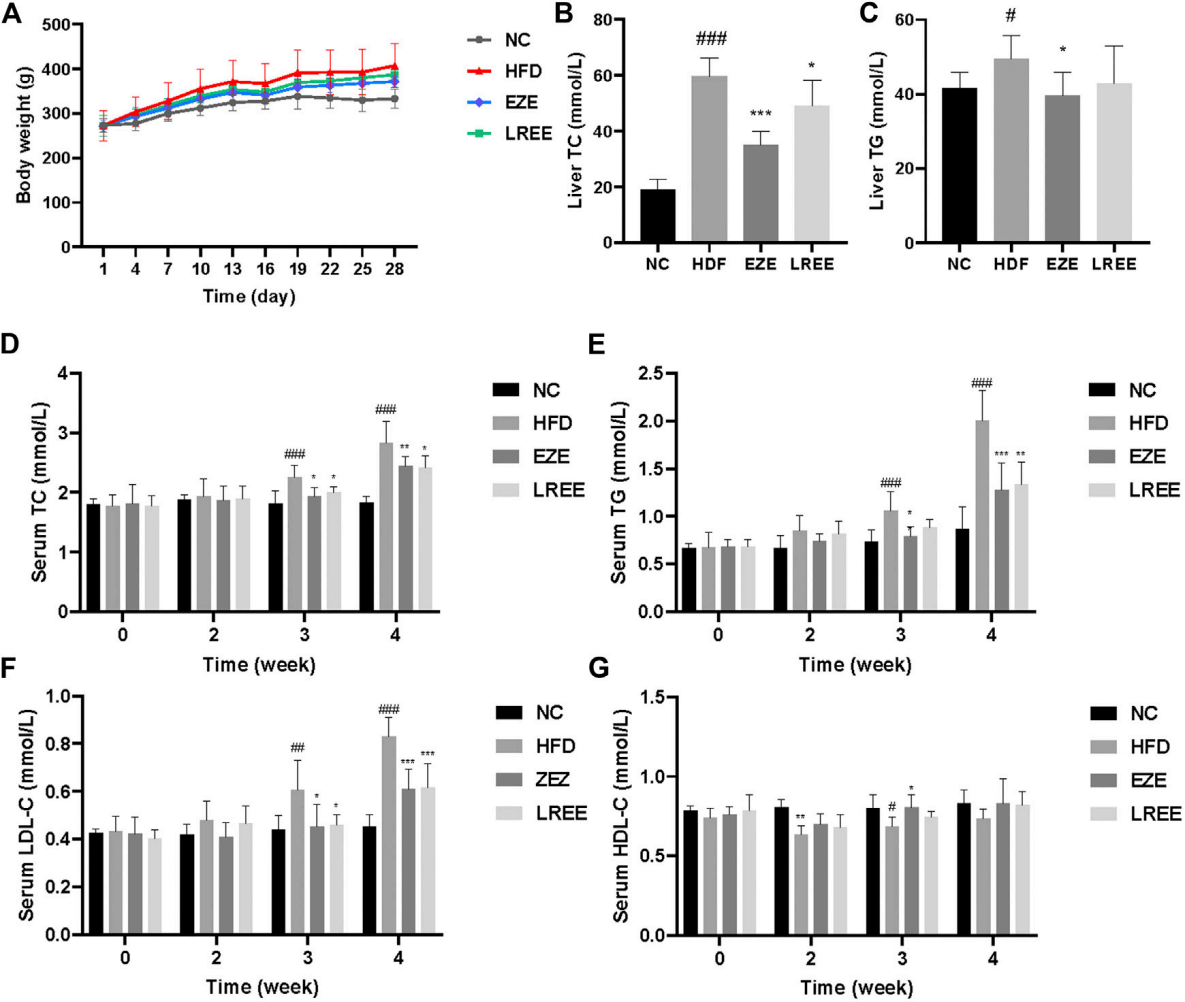
Effects of LREE on HFD-induced hyperlipidemia in rats. **(A)** Body weight during modeling time. **(B)** TC levels in the liver. **(C)** TG levels in the liver. **(D)** TC levels in the serum. **(E)** TG levels in the serum. **(F)** LDL-C levels in the serum. **(G)** HDL-C levels in the serum. ^#^ *p* < 0.05, ^##^ *p* < 0.01, ^###^ *p* < 0.001 via the NC group; * *p* < 0.05, ** *p* < 0.01, *** *p* < 0.001via HFD group.

A high-fat diet was used to construct a hyperlipidemia rat model. Dynamic measurements of body weight showed that a high-fat diet could significantly lead to additional weight gain in rats (Figure 2A). Besides that, high-fat diet also significantly caused the elevation of liver weight and liver coefficient, which could be reduced by LREE. On food intake, a high-fat diet resulted in a decrease in food intake in rats, which was not significantly affected by EZE and LREE (Table 2). TC and TG were sensitive markers for hyperlipidemia. Compared with the normal diet rats, a high-fat diet caused a rise in liver TC and TG levels (Figures 2B,C). Continuous observation in serum proved that the TC and TG levels in the HFD group significantly increased after 3 weeks of special feeding. Both LREE and EZE treatment could rectify these changes and EZE showed better treatment effects (Figure 2D,E). We also measured the HDL-C and LDL-C in different periods. A high-fat diet can promote serum LDL-C level that can be blocked by EZE and LREE (Figure 2F). Compared to LDL-C, there are no significant changes in HDL-C but a slight trend was found in both treatment groups (Figure 2G). These data suggest that LREE improves hyperlipidemia mainly by regulating serum TC and LDL-C levels.

**TABLE 2:** Effect of LREE on the body weight, body composition, and food intake in rats fed with HFD.

Item	Groups			
	NC	HFD	EZE	LREE
Final BW(g)	333.33 ± 21.98	407.08 ± 49.66^a^	371.43 ± 11.29	386.75 ± 11.20
Food intake (g/d)	23.09 ± 1.41	18.23 ± 2.19^a^	18.58 ± 4.78	20.08 ± 3.64
Liver(g)	8.75 ± 1.19	17.09 ± 1.58^a^	11.84 ± 0.90^b^	14.84 ± 0.52^b^
Liver coefficient	2.63 ± 0.40	4.24 ± 0.66^a^	3.19 ± 0.26^b^	3.84 ± 0.09

^a:^ compare to NC group, *p*<0.01

^b:^ compare to HFD group, *p*<0.01.

### LREE Alleviate Liver Injury in Hyperlipidemia Rats

**FIGURE 3:**
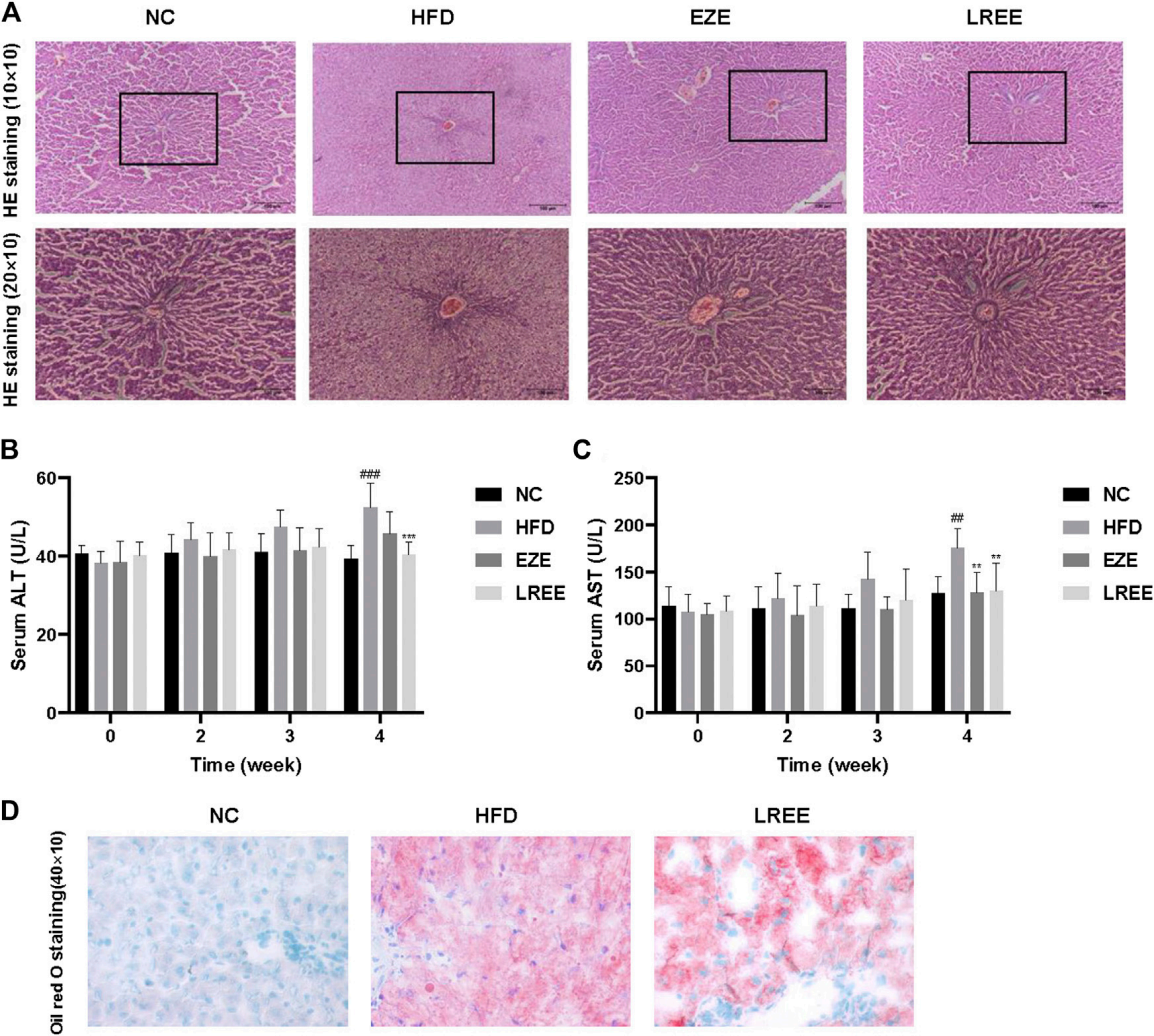
Effects of LREE on HFD-induced liver injury in rats. **(A)** Representative images of liver histopathology (Hematoxylin and eosin staining). Normal liver tissue structure is complete, hepatocytes are arranged neatly, with uniform size and no steatosis. In the HFD group, the hepatocytes in the liver tissues of rats were disordered, with severe steatosis and a large number of fat vacuoles. Liver tissue structure was intact and fat vacuoles were significantly reduced in EZE and LREE treatment groups. **(B)** ALT levels in the serum. **(C)** AST levels in the serum. **(D)** Oil red O staining of livers from HFD group and LREE group rats. ^##^ *p* < 0.01, ^###^ *p* < 0.001 via the NC group; ** *p* < 0.01, *** *p* < 0.001 via HFD group.

High fat and high cholesterol diet are triggers resulting in fatty liver. To assess whether LREE can protect against liver damage caused by high lipids, we checked the pathology changes in the liver. H&E staining results showed extensive steatosis and hepatocyte disordered in HFD rat’s liver, and LREE could inhibit these changes caused by a high-fat diet (Figure 3A). We also measured the levels of ALT and AST in serum to evaluate the degree of hepatocyte damage. The result showed that compared to the NC group, the ALT and AST activity of model rats were significantly upregulated at 4 weeks. EZE and LREE treatment reduced both ALT and AST activity while LREE works better in reducing ALT (Figure 3B,C). We also evaluated the improvement of hepatic lipids by LREE using oil red O staining. As a result in Figure 3D, LREE reduced lipid deposits on the liver. These data suggested that LREE may have better liver protection and this provides more possibilities for LREE as an anti-hyperlipidemia drug.

### LREE Reverses Intestinal Flora Disorder in Hyperlipidemia Rats

**FIGURE 4:**
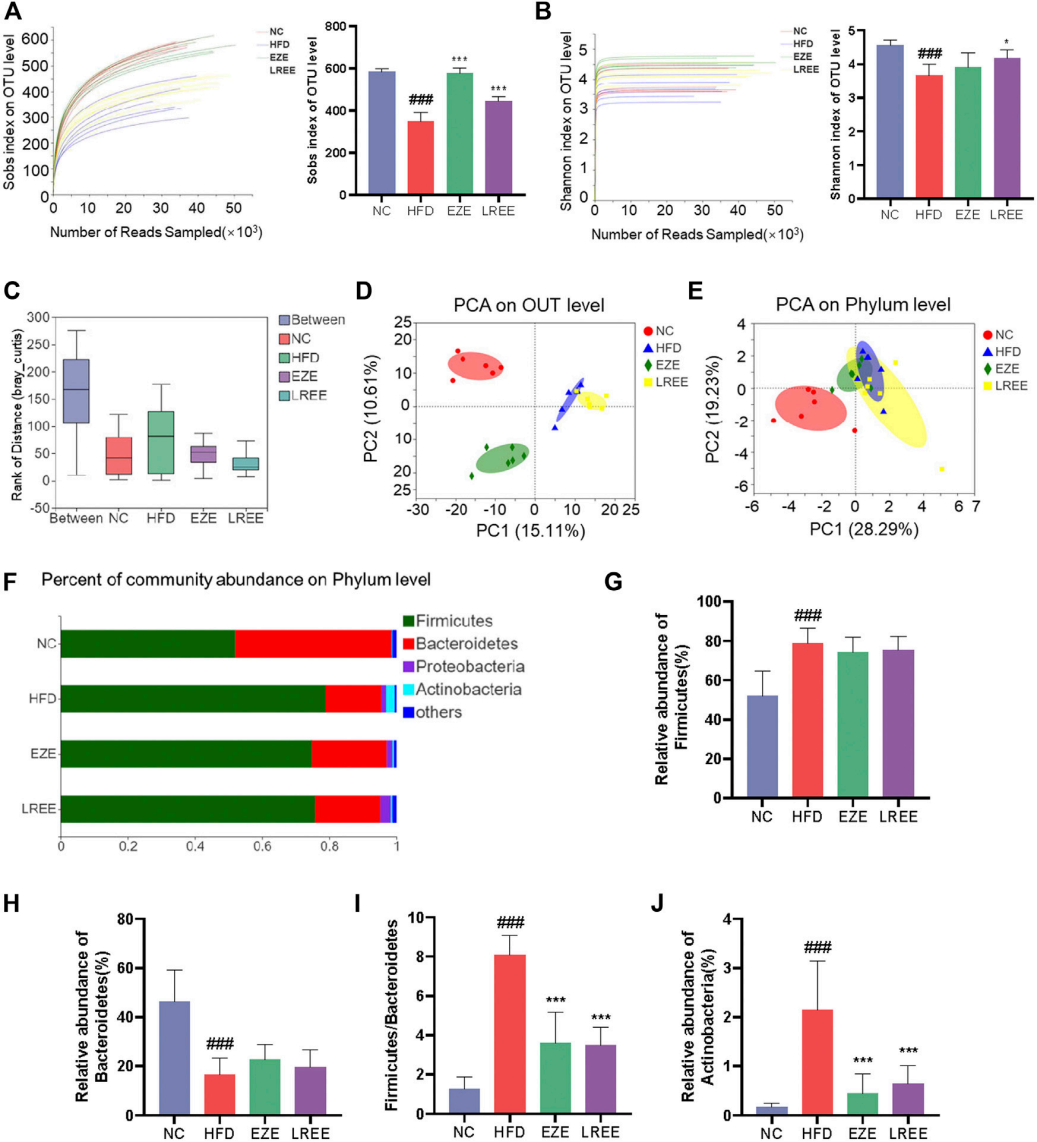
Diversity analysis for bacteria in diverse groups. Sobs index **(A)** and Simpson index **(B)** rarefaction curve and the OUT level **(C)** ANOSIM/Adonis analysis of four groups. **(D)** PCA analysis of four groups in OUT level. **(E)** PCA analysis of four groups in phylum level. **(F)** The proportion of major bacteria in phylum level and detail abundance of firmicutes **(G)**, Bacteroidetes **(H)**, the ration of F/B **(I)**, and Actinobacteria **(J)**. ^###^ *p* < 0.001 via the NC group; * *p* < 0.05, ** *p* < 0.01, *** *p* < 0.001via HFD group.

Gut microbiota plays an important role in metabolic diseases. Drugs, especially those oral administrated, often have a huge impact on microbiota composition. Therefore, we studied the effects of a high-fat diet on the microbiota and the regulation of LREE. A total of 1184130 raw data was gained by 16s RNA sequencing with an average of 49,338.75 per sample. Sobs index and Shannon index were used to evaluate the OUT abundance of each group. The dissolution curve shows that an in-depth sequencing was performed in each group and can be used for further analysis (Figure 4A,B). The results showed a significant difference between the four groups while a high-fat diet can cause a decrease in the OTU level and LREE can recover the OTU abundance (Figure 4A,B). This suggested that hyperlipidemia reduces the abundance of gut microbiota, leading to host dysfunction. We further analyzed the effects of hyperlipidemia on the internal composition of the gut microbiota. Similarities analysis (ANOSIM) and principal analysis (PCA) were performed to compare the similarity between the four groups. The result indicated that intragroup differences were much smaller than the difference between the group with an R = 0.8035 (Figure 4C) and PCA indicate a significant difference in OTU level and phylum level (Figure 4D,E). On phylum level, Firmicutes and Bacteroidetes are the main composition of each group (Figure 4F). Detailed composition indicated that a marked increase of Firmicutes and a decrease of Bacteroidetes was closely related to hyperlipidemia (Figure 4G,H). LREE treatment has a tendency to restore their composition but no significant difference was found. As the bacteria with the highest relative abundance, the balance of Firmicutes and Bacteroidetes is of great significance for maintaining intestinal homeostasis. The results showed that LREE could restore F/B balance significantly (Figure 4I). In addition, an abnormal elevation of actinobacteria was found in the HFD group. This abnormal bacterial enrichment was significantly inhibited after LREE treatment (Figure 4J). The above results indicated that gut microbiota disturbance in hyperlipidemia rats may be one of the reasons for its pathogenesis, and LREE can repair disturbed flora homeostasis to some extent.

### LREE treats hyperlipidemia by altering bile acid metabolism mediated by gut microbiota.

**FIGURE 5:**
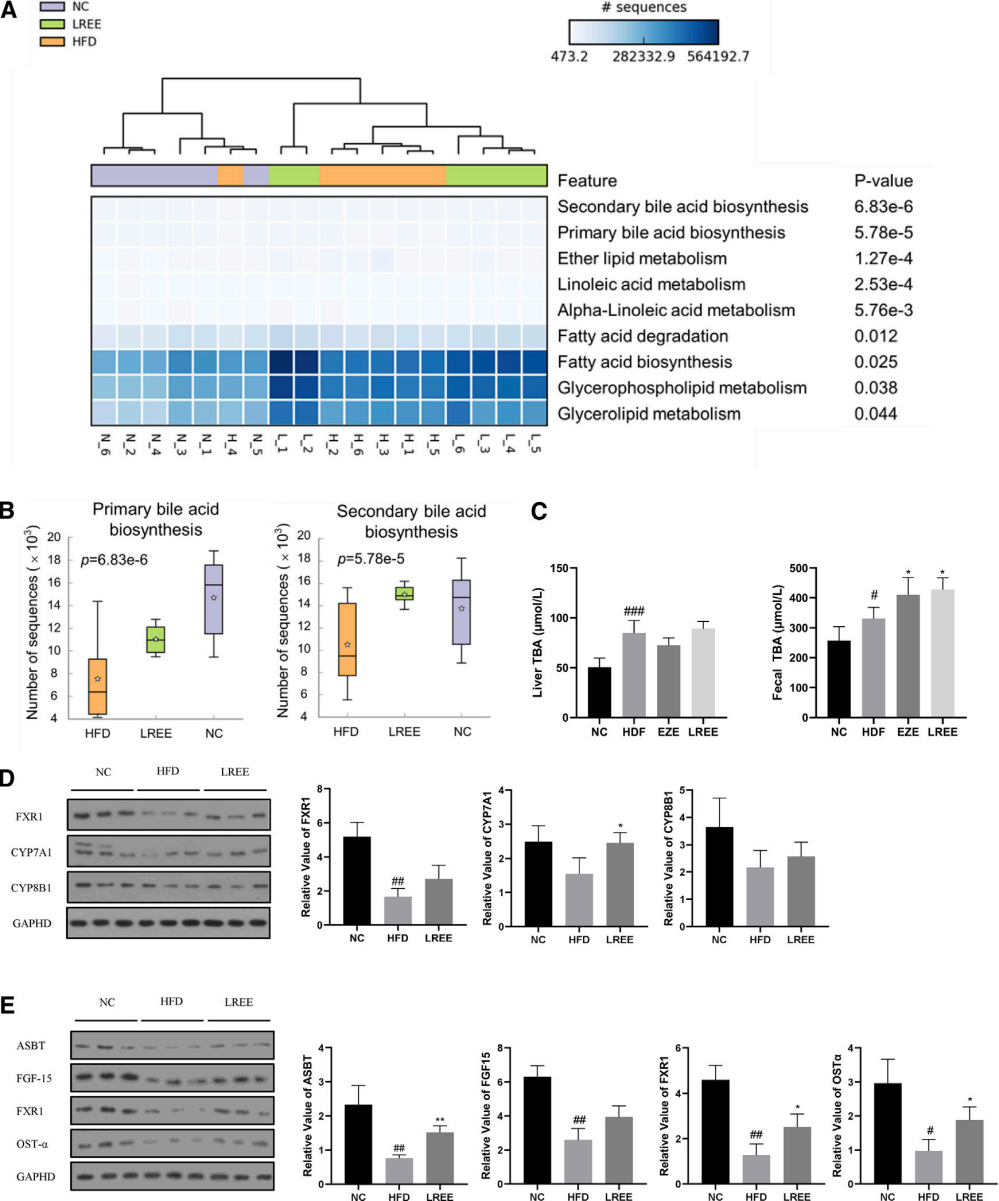
**(A)** Enrichment analysis of the metabolic function of intestinal flora in three groups. The darker colors in the cells suggest that more microbiota was involved. The top ranking on the right indicates a more significant difference between the groups. **(B)** The abundance of bacterial flora involved in bile acid synthesis between different groups. **(C)** Levels of total bile acids in liver and feces. **(D)** Expression of the major proteins involved in primary bile acid synthesis in the liver. **(E)** Expression of major proteins involved in bile acid reabsorption in the ileum. ^#^ *p* < 0.01, ^##^ *p* < 0.01, ^###^ *p* < 0.001 via the NC group; * *p* < 0.05, ** *p* < 0.01 via HFD group.

Gut microbiota can alter host physiology through multiple metabolites, and we used PICRUSt2 to predict these metabolic changes that may play a central role in hyperlipidemia. The analysis showed that various metabolic processes, including bile acid metabolism, fatty acid metabolism, and glucose metabolism were associated with microbiota. Among them, the most significant difference was found in bile acid metabolism, including the synthesis of primary and secondary bile acids (Figure 5A). Microbiota analysis in the HFD group showed that the synthesis levels of primary and secondary bile acids were reduced, while this was significantly improved after LREE treatment (Figure 5B). To validate this result, we measured total bile acid levels in the livers and feces of rats, and the results showed that HFD led to an increase in liver bile acid levels and LREE led to an increase in fecal bile acid excretion (Figure 5C). Detailed analysis showed that a high-fat diet resulted in abnormalities of both primary and secondary bile acids (Table 3). After LREE treatment, the levels of multiple primary bile acids including TCA, TUDCA in serum were increased and the levels of multiple secondary bile acids were significantly decreased (Table 4). This implied that, as predicted, bile acid metabolism is disturbed in HFD-induced hyperlipidemic rats, and LREE has a regulatory effect on bile acid metabolism. LREE affected the metabolites and accelerated the synthesis of primary bile acids and excretion of secondary bile acids.

**TABLE 3:** Differential bile acids between normal groups and high-fat diet groups.

Class	Metabolite	NC	HFD	Log2FC	*p*-value
Primary BAs	TCA	246.07 ± 94.52	111.57 ± 38.72	−1.40	7.93651E-03
Primary BAs	UDCA	171.33 ± 86.28	1,417.26 ± 633.01	2.86	7.93651E-03
Primary BAs	CDCA	2605.00 ± 2070.08	5339.15 ± 2651.7	1.16	3.17460E-02
Secondary BAs	THDCA	772.35 ± 292.19	75.68 ± 49.80	−3.32	7.93651E-03
Secondary BAs	βCA	13.89 ± 9.83	42.03 ± 19.92	1.66	3.17460E-02
Secondary BAs	7-KetoLCA	192.65 ± 200.84	538.00 ± 265.10	1.66	3.71338E-02

TCA, taurocholic acid; UDCA, ursodeoxycholic acid; CDCA, chenodeoxycholic acid; THDCA, taurohyodeoxycholic acid; βCA, 3β-Cholic Acid; 7-ketoLCA, 7-ketolithocholic acid.

**TABLE 4:** Differential bile acids between HFD groups and LREE treatment groups.

Class	Metabolite	HFD	LREE	Log2FC	*p*-value
Primary BAs	TUDCA	5.65 ± 1.30	7.59 ± 1.41	0.42	2.64000E-02
Primary BAs	TαMCA	134.22 ± 31.83	216.88 ± 79.74	0.70	3.18000E-02
Primary BAs	TβMCA	54.71 ± 19.61	95.17 ± 37.15	0.80	3.17000E-02
Primary BAs	TCA	111.57 ± 38.72	169.25 ± 51.49	0.60	4.01000E-02
Primary BAs	TCDCA	39.66 ± 9.99	61.58 ± 24.51	0.63	5.06000E-02
Secondary BAs	HDCA	1,494.89 ± 849.74	314.95 ± 253.42	-2.25	8.86000E-03
Secondary BAs	THDCA	75.68 ± 49.8	17.21 ± 6.51	-2.12	1.57000E-02
Secondary BAs	TωMCA	29.94 ± 13.12	13.93 ± 7.06	-1.09	2.15000E-02
Secondary BAs	6-KetoLCA	142.07 ± 85.37	42.11 ± 42.35	-1.74	5.06000E-02

TUDCA, tauroursodeoxycholic acid; TαMCA, tauro α-muricholate; TβMCA, tauro β-muricholate; TCA, taurocholic acid; TCDCA, taurochenodeoxycholate; HDCA, α-hyodeoxycholic acid; THDCA, taurohyodeoxycholic acid; TωMCA, tauro ω-muricholate; 6-ketoLCA, 6-ketolithocholic acid.

To further investigate how microbiota alters bile acid metabolism, we examined the expression levels of genes closely related to bile acid synthesis and reabsorption. Compared with the normal rats, genes involved in the synthesis of bile acids in the liver, including CYP7A1 and CYP8B1, were not significantly affected by a high-fat diet, but nuclear farnesoid X receptor (FXR) was significantly downregulated (Figure 5D). Compared with the HFD group, LREE only increased the expression of CYP7A1, which indicated that LREE might be involved in the synthesis of some bile acids and lead to the elevation of bile acid synthesis (Figure 5D). In addition to these minor changes, it is more likely that microbiota alteration readjusts the reabsorption of bile acids by affecting the production of secondary bile acids. Under normal conditions, intestinal bile acid reabsorption is mainly mediated by ASBT and OST-α (Ballatori et al., 2005). Gut microbiota can directly regulate ASBT expression through transcription factors (Out et al., 2015). In addition, secondary bile acids produced by gut microbiota also regulates the expression of FXR in the intestine through bile salt hydrolase (BSH) genes, leading to changes in ASBT to affect the bile acid reabsorption. The reabsorbed bile acids regulate the further synthesis of bile acids in the liver through FGF-15 (Kliewer and Mangelsdorf, 2015). Together, these cascade reactions caused by microbiota maintain normal bile acid metabolism in the body. Our results showed that the expression of bile acid reabsorption-related proteins including ASBT, OST-α, and FXR in the ileum decreased markedly in hyperlipidemia rats (Figure 5E). This means that bile acid reabsorption is greatly weakened, while LREE restores some of the bile acid reabsorption function. However, there was no significant change in FGF-15 after LREE treatment, suggesting that the ability of LREE to affect hepatic bile acid production through microbiota is slight. Combined with the levels of hepatic and fecal bile acids in Figure 5C, we speculate that LREE plays a therapeutic role in hyperlipidemia mainly by altering the gut microbiota and repairing the bile acid reabsorption disrupted by HFD.

## Discussion

In this study, we established a hyperlipidemia model and used LREE to deliver the intervention. The results revealed that LREE can regulate serum TC and LDL-C levels to play an anti-hyperlipidemic role. Compare to the classic drug, LREE has a weak lipid reduction but better liver protection. This is inspiring as liver injury caused by high-lipid and high-cholesterol was frequently observed in clinical practice. At present, the application of a single drug for hyperlipidemia, such as statin and ezetimibe, has been limited because of the inadequate control of lipids and side effects. Therefore, combination-drugs has become a promising direction to improve clinical efficacy. For instance, ezetimibe-statin combination is a more efficacy and safe therapy in clinical treating (Nußbaumer et al., 2016). Unfortunately, numerous adverse effects are still a matter of concern (Ferri and Corsini, 2020). The hypolipidemic and hepatoprotective properties of LREE make it a potential candidate for hyperlipidemia treatment or combination therapy.

Except for the clinical effect of LREE, we turned our attention to the mechanism of LREE treatment. Gut microbiota alterations are known to contribute to metabolic diseases (Cani, 2019). Gut microbiota can generate a large number of metabolites that can transfer to liver or blood to regulate metabolism. In the regulation of microbiota, diversity is one of the important dimensions to ensure normal intestinal function. In the present study, a decrease in OUT level was first observed in high-fat diet feed rats which agreed with the current studies. Many metabolic diseases including diabetes, hypercholesterolemia, and fatty liver show an extreme reduction of gut microbiota (Zhao et al., 2018). Similarly, our results suggested that hyperlipidemia is also closely associated with a reduction in gut microbiota diversity and this provided evidence for the treatment of hyperlipidemia by improving the gut microbiota.

Firmicutes and Bacteroidetes are the two most abundant bacteria known in the gut, and more than 90% of the intestinal flora belongs to them (Qin et al., 2010). Both Firmicutes and Bacteroidetes play a pivotal role in keeping metabolic balance in the human body. The ratio of Firmicutes/Bacteroidetes (F/B) was an important signal for microbiota steady. Multiple studies have confirmed that a higher F/B ratio might be significant indicators for obesity (Koliada et al., 2017; Indiani et al., 2018; Magne et al., 2020). Some studies have revealed that reversed the F/B ratio is helpful for hyperlipidemia treatment (Wang et al., 2019). Not only that, Bacteroidetes are thought to be beneficial to health as they can exhibit specific enzymes to degrade non-digestible carbohydrates and modulate calorie absorption (Gibiino et al., 2018). Excess energy intake was closely related to reduced numbers of Bacteroides (Simoes et al., 2013). Likewise, in our study, Bacteroidetes was found extremely reduced while Firmicutes was found to be significantly increased in hyperlipidemia rats. Although LREE only shows a trend to adjust the abundance of Firmicutes or Bacteroidetes, significant differences in the ratio of F/B were observed in LREE treating group. This may be the mechanism by which LREE intervenes in hyperlipidemia. In addition to these two representative bacteria, actinobacteria were also found a marked change in different groups. As a minority of gut microbiota, actinobacteria was a controversial flora and some researches indicate a good effect for human health (Cani et al., 2007) while another research indicates that actinobacteria was positively associated with fat but negatively associated with fiber (Wu et al., 2011). In this study, a risen actinobacteria was found increased in rats feed with high-fat diet and LREE can reduce it. Therefore, based on the evidence, actinobacteria may play a driving role in the development of hyperlipidemia.

Alterations in gut microbiota can cause a series of functional changes, especially metabolic changes.

Bile acid metabolism is one of the most immense metabolic processes affected by gut microbiota and is also a key pathway of cholesterol metabolism *in vivo*. Bile acids are produced in the liver from cholesterol and, subsequently, processed into secondary bile acids by the gut microbiota. Bile acid synthesis and excretion are regulated by FXR and several rate-limiting enzymes in the liver (Lee et al., 2006). The classical pathway is mainly involved by CYP7A1 and CYP8B1, and about 75% of primary bile acids are synthesized by this pathway (Russell, 2003). The activity of CYP7A1 is negatively regulated by the FXR/FGF15 axis (Kliewer and Mangelsdorf, 2015). In our study, FXR in the intestine was extremely reduced in hyperlipidemia rats, which would relieve the inhibition of CYP7A1 and thus produce more primary bile acids in liver.

Consistent with the results of Figure 5C, we found a significant increase in bile acids in the liver of hyperlipidemia rats. The increase in bile acids enlarges the contact area of emulsified fat with lipase and promotes intestinal fat transport, thereby promoting fat absorption. In addition, multiple bile acids including primary bile acids and secondary bile acids were found to be up-regulated in hyperlipidemia rats serum. Among them, the increased primary bile acids were mainly, UDCA and CDCA. UDCA (Ceryak et al., 2000) and CDCA (De Leon et al., 1979) are both considered center bile acids for the improvement of hyperlipidemia. In hyperlipidemic rats, disruption of bile acid metabolism leads to massive accumulation of these two major bile acids in the serum, which will further lead to the lipid metabolic disorders in intestine. More importantly, excessive synthesis of bile acids in hyperlipidemia rats do not enhance the intestinal reabsorption of bile acids. Intestinal reabsorption is essential for maintaining the circulation of bile acids, which is mainly mediated by ASBT, OST-α, and OST-β (Ballatori et al., 2005). In hyperlipidemic rats, ASBT and OST-α in the intestine are significantly reduced. So too much bile acid accumulates in the intestine to accelerate the digestion and absorption of fat acid, which is responsible for the occurrence of hyperlipidemia and also possible to promote intestinal inflammation. Our results suggested that LREE can reverse the impaired bile acid metabolism and restore the expression of related proteins. We sought to identify the bacterium responsible for these actions. In the small intestine, microbiota enriched with BSHs functions for the deconjugation of conjugated bile acids (Jones et al., 2008), which is followed by the production of secondary bile acids by the microbiota. This is particularly important for the whole bile acid metabolism, as secondary bile acids are ligands for FXR, mediating the activation and inhibition of FXR, thereby affecting metabolic cascade reactions. In our study, the increasing of the fecal bile acid excretion also confirmed a acceleration in synthesis of secondary bile acids. Based on metagenomic screening, three major bacterium have BSHs: Firmicutes (30%), Bacteroidetes (14.4%), and Actinobacteria (8.9%) (Jones et al., 2008). Coincidentally, this is consistent with the results obtained in our above studies. Hyperlipidemia results in a reduction in Bacteroidetes, and a elevation in Firmicutes and Actinobacteria. After LREE treatment, the alterations were reversed, along with the reabsorption of bile acids damaged by HFD. Further analysis also demonstrated that LREE reduced the levels of varies secondary bile acids in serum. LREE promotes the unconjugation of primary bile acid by gut microbiota and accelerates its excretion. These produced secondary bile acids also act as ligands to further stimulate the synthesis of primary bile acids, thereby accelerating cholesterol transport and efflux. All these results strongly suggested that LREE can accelerate bile acids metabolism by regulating the composition of Firmicutes, Bacteroidetes, and Actinobacteria to exert an anti-hyperlipidemia effect.

**FIGURE 6:**
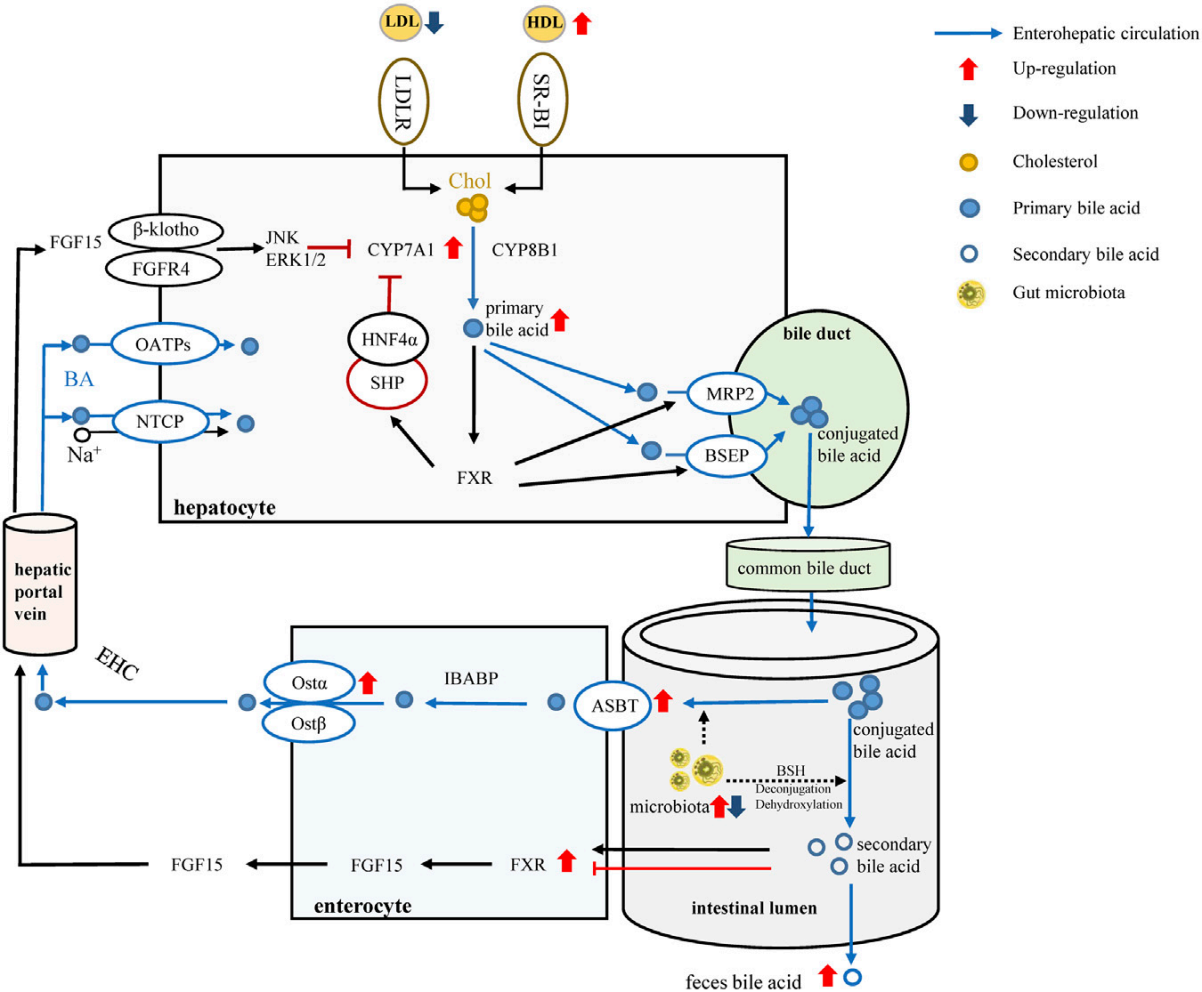
LREE regulates bile acid metabolism through intestinal flora. Red arrows and blue arrows indicate up-regulation and down-regulation after LREE treatment. Normally, most of the primary bile acids are produced by cholesterol through the classical metabolic pathway involving CYP7A1 and CYP8B1. Primary bile acids form conjugated bile acids through the bile duct and are discharged into the intestine. Most of the conjugated bile acids are reabsorbed by the ASBT/OST system and participate in the enterohepatic circulation of bile acids. A small proportion of conjugated bile acids are deconjugated and dehydroxylated by microbiota, converting to secondary bile acids. Different secondary bile acids can activate or inhibit FXR and lead to the release of FGF15. Subsequently, FGF15 entered the liver via the hepatic portal vein to inhibit the expression of CYP7A1 and maintain bile acid levels together with the reabsorbed bile acids. LREE can restore the expression of ASBT, OST-α, FXR, CYP7A1 affected by hyperlipidemia, restore the reabsorption of bile acids, and promote the conversion of cholesterol into bile acids. Meanwhile, LREE increases the synthesis of secondary bile acids and accelerates their excretion through intestinal flora. Increased bile acid metabolism will increase the rate of lipid metabolism and maintain lipid homeostasis in the body.

In conclusion, our study confirmed that LREE can be an effective drug for hyperlipidemia. It can regulate lipid metabolism and alleviate liver injury. LREE can greatly affect gut microbiota altered by a high-fat diet thus affecting bile acid metabolism (Figure 6). Despite the significance of our findings, our study has certain limitations. We are unable to provided sufficient evidence to prove that alterations in gut microbiota directly affect bile acid metabolism. To this purpose, the design of individual flora therapy experiments is necessary in subsequent studies. Even so, our study provides some new insights into the treatment of hyperlipidemia from the perspect of flora, and we also provide some evidence for the selection of natural drugs in hyperlipidemia treatment.

## Data Availability

The datasets presented in this study can be found in online repositories. The name of the repository and accession number can be found below: National Genomics Data Center, https://bigd.big.ac.cn/, CRA003502.
